# Cervical dysplasia in elderly women performing repeated self-sampling for HPV testing

**DOI:** 10.1371/journal.pone.0207714

**Published:** 2018-12-05

**Authors:** Annika Kristina Lindström, Ruth Sanchez Hermansson, Inger Gustavsson, Julia Hedlund Lindberg, Ulf Gyllensten, Matts Olovsson

**Affiliations:** 1 Department of Women´s and Children’s Health, Uppsala University, Uppsala, Sweden; 2 Center for Clinical Research, Dalarna, Uppsala University, Uppsala, Sweden; 3 Clinical Research Center, Faculty of Medicine and Health, Örebro University, Örebro, Sweden; 4 Department of Oncology, Faculty of Medicine and Health, Örebro University, Örebro, Sweden; 5 Department of Immunology, Genetics and Pathology, Uppsala University, Uppsala, Sweden; Albert Einstein College of Medicine, UNITED STATES

## Abstract

**Background:**

About 30% of the cervical cancer cases in Sweden occur in women older than 60. The primary aim was to evaluate the acceptability of repeated self-sampling at home for HPV-testing in elderly women. The prevalence of HPV and HPV related dysplasia as well as the sensitivity of cytology was evaluated.

**Methods:**

Repeated self-sampling at home for HPV testing was offered 375 women in each of the four age groups 60, 65, 70 and 75 years. Women with two consecutive positive HPV tests were examined with sampling for histology and cytology.

**Findings:**

A self-sample was provided by 59.5% (893/1500) of the invited women. The overall prevalence of HPV was 4.4% (95% CI 3.2–6.0, n = 39) in the first test, and 2.5% were persistent positive in the second test (95% C 1.6–3.8, n = 22) collected on average 5.5 months later. Dysplasia, was found in 1.8% (16/893) (95% CI 1.1–3.0) and CIN 2+ in 1.0% (9/893) (95%CI 0.5–2.0) of the women. Of the 16 women with dysplasia in histology, 13 (81.2%) had a normal cytology.

**Interpretation:**

Repeated self-sampling at home combined with HPV testing was well accepted among elderly women. A high prevalence of CIN was diagnosed by histology. Cytology showed extremely low sensitivity and should not be recommended for this age group.

## Introduction

Despite a high incidence of cervical cancer (CC) in women over the age of 60, elderly women are not included in the screening programmes for cervical cancer prevention. In Sweden, about 30% of CC cases occur in women over 60 and the mortality rate is about 70% in this age group [1–3]. Cervical cancer in women above the age of 65 is usually discovered at advanced stages and the prognosis is poor [4]. A combination of organised and opportunistic Pap smear screening has reduced the incidence of squamous cell cancer in cohorts most regularly screened, by around 70% [5]. The Swedish screening programme that was used during the study period ended at the age of 60. The 2015 Swedish cervical screening guidelines (www.socialstyrelsen.se) are under implementation and from 2017 HPV based screening for women of 30–64 years of age is recommended [6]. During the past century, the average life expectancy for Swedish women has increased from 55 to 84 years, and many women over 65 are healthy [7], continue to work, and have an active sex life [8].

It is well known that DNA based HPV tests are more sensitive than cytology in detecting pre-malignant lesions on the cervix [9–11]. Implementation of an HPV test in screening programmes is therefore recommended [9, 10]. It is also well known that a single HPV test is associated with a modest specificity that can be significantly improved with repeated testing [11, 12]. Vaginal self-sampling for HPV testing has been extensively studied, and results from self-sampling are fully comparable with samples collected by a healthcare provider (HCP), as long as a sensitive PCR-based method is used [13, 14].

In post-menopausal women, due to hormonal changes, the transformation zone where precursor lesions develop is situated higher up in the cervical canal, and is therefore not accessible for proper examination and sampling [15]. Cytology and colposcopy are thus less appropriate methods for the detection of cervical dysplasia in this age group [16].

In elderly women, there is a lack of knowledge concerning the prevalence of HPV and HPV related dysplasia. There are few studies focusing on women who are older than those included in the screening programmes [17]. In a recent study, we have shown that a significant proportion of elderly women have a persistent cervical HPV infection and a high prevalence of CIN diagnosed by histology. It was also shown that cytology has extremely low sensitivity in this age group [17]. There are however no studies on self-sampling in elderly women.

The primary aim of the current study, therefore, was to evaluate the acceptability of repeated self-sampling for HPV testing in elderly women. The prevalence of HPV and HPV related dysplasia, as well as the sensitivity of cytology, was also evaluated.

## Materials and methods

**Fig 1:**
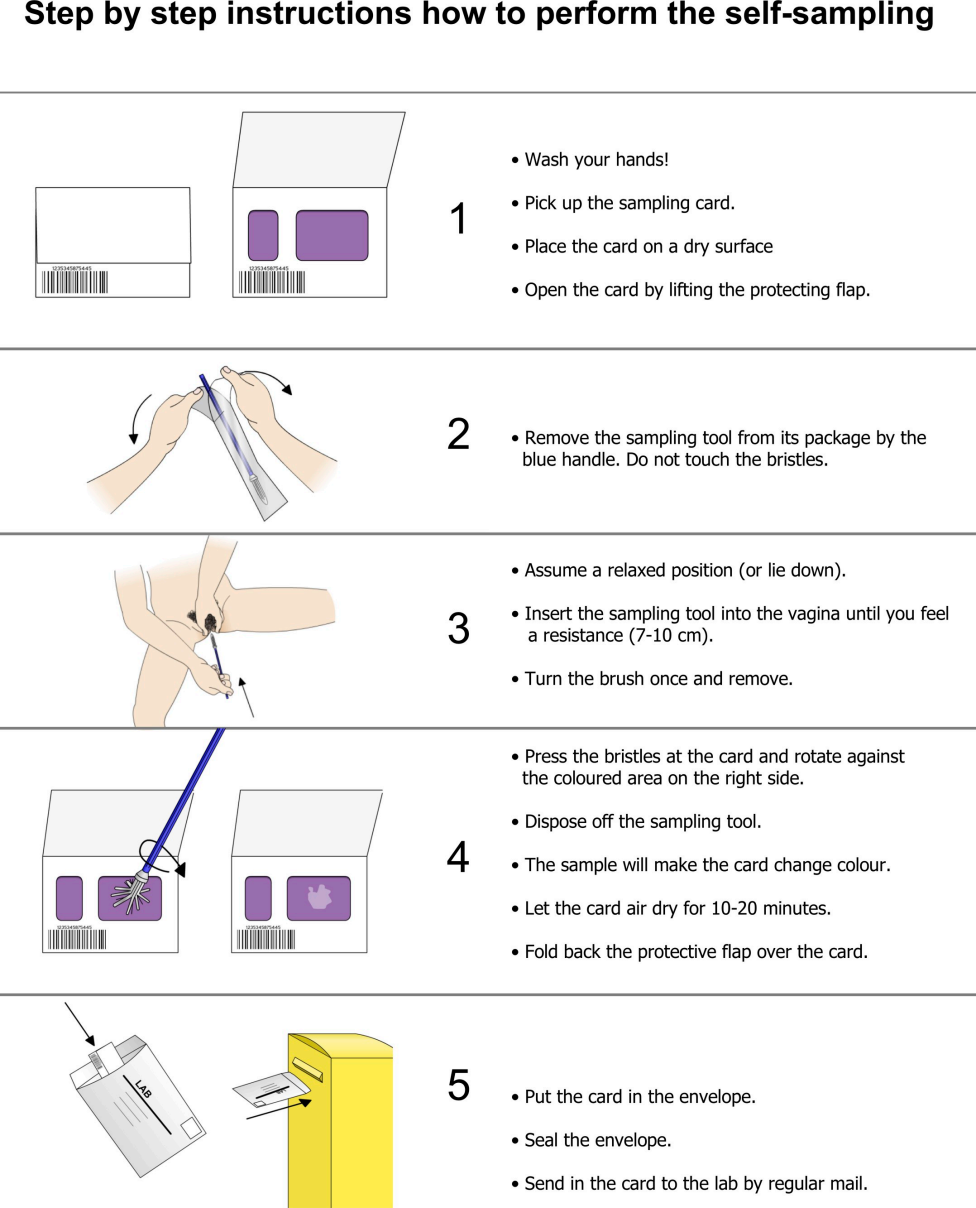
Information supplied to the women on how to perform the self-sampling using the FTA card.

This prospective population based longitudinal descriptive study was conducted in Dalarna County, Sweden, between 2014 and 2016, and 1 500 women were invited to perform repeated self-sampling for HPV testing. Women were randomly selected, by using the random generator in SPSS, from the population register, with 375 women in each of the four age groups 60, 65, 70 and 75. Written informed consent was obtained from women who agreed to participate in the study, and self-sampling kits were sent through the postal service. In brief, self-sampling was performed at home (Fig 1) and the sample was returned, in a prepaid envelope, to the laboratory for analysis of high-risk HPV, as previously described [12]. Women with a positive first HPV test were sent a new self-sampling kit four months after the first test was done, as previously described [11]. The HPV test was done using a multiplex real-time PCR assay (hpVIR), as earlier described [18], which detects the high-risk HPV types 16, 18, 31, 33, 35, 39, 45, 51, 52, 56, 58 and 59 (18 and 45 are detected together, and 33, 52 and 58 as one group). A cervical sample was collected using the RoversViba-brush (Rover Medical Devices B.V., Oss, The Netherlands). The sample was applied to a filter paper matrix, an indicating FTA elute card (GE Healthcare, Cardiff, UK art. no WB129308), and the DNA was obtained from the FTA cards as described earlier [19]. The threshold for a positive HPV-type was set to 10 copies per PCR [18]. The distribution of kit and HPV typing was undertaken by the HPV laboratory, Department of Immunology, Genetics and Pathology, Uppsala University, Uppsala, Sweden.

Women who were repeatedly positive in the second HPV test were offered examination by colposcopy, sampling for histology, and liquid-based cytology (LBC). One of the authors (RSH) performed the vast majority of the colposcopies, cervical biopsies, abrasions, and conisations for histological diagnosis.

All LBC specimens were screened by cytotechnicians, and those considered abnormal were reviewed by a surgical pathologist. For cervical cytology, the Thin Prep Pap Test was used. The cervical smear was collected with a plastic spatula and a cytobrush. LBC specimens were placed in PreserveCyt solution and processed in the Thin Prep 5000-processor (HologicCytyc Corporation, Boxborough, Mass.) [20]. The terminology for classification of cytology was for squamous cell lesions categorised into atypical squamous cells of undetermined significance (ASCUS), atypical squamous cells high-grade squamous lesion cannot be excluded (ASC-H), and cervical intraepithelial neoplasia grade 1–3 (CIN 1–3). For histology, the CIN classification was used.

Specialists in surgical pathology examined the cervical biopsy samples and cones for histologic diagnosis. One senior pathologist re-evaluated all LBCs, cervical biopsies and cones, focusing on glandular atypia and adenocarcinoma. All cytology and histology specimens were examined at the Department of Pathology and Cytology, Falun County Hospital, Falun, Sweden.

For statistical analysis, Excel and the Statistical Package for Social Sciences (SPSS) version 22 for Windows were used. The data was analysed using both a per-protocol approach (PP), including only women who complied with the protocol, and also with an intention-to-treat approach (ITT), by also including women who on their own initiative had a clinical second HPV test and follow up. A p-value less than 0.05 was considered statistically significant. For statistical significance testing between age groups, the chi-squared test in SPSS was used. Confidence intervals (CI) of proportions (Fleiss) were calculated using Excel [21]. The study was approved by the Regional Ethics Committee in Uppsala (Dnr 2014/024).

## Results

**Fig 2:**
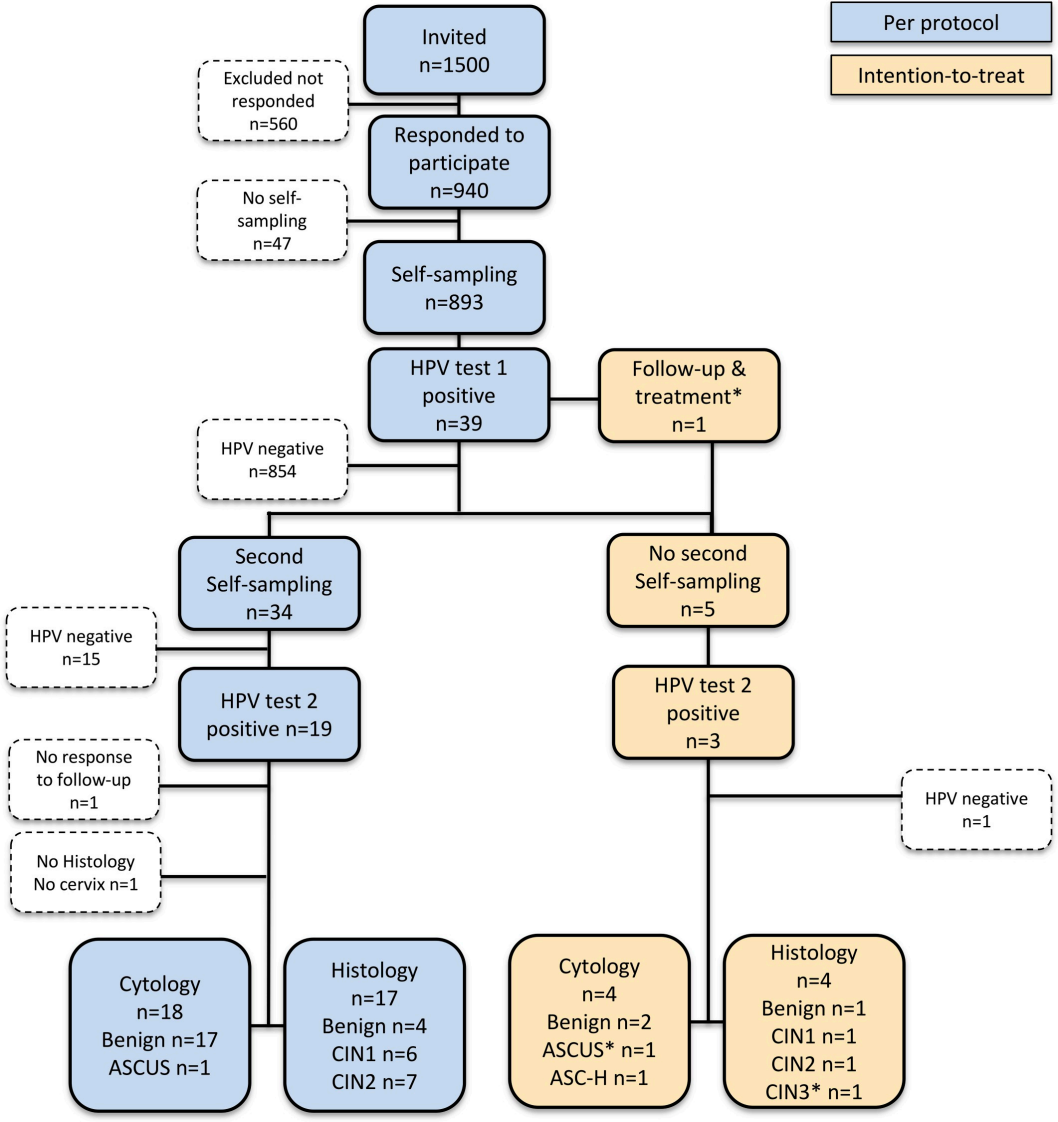
Flowchart showing study design and HPV and dysplasia occurrence (n = 893). HPV (human papillomavirus) ASCUS (atypical squamous cells of undetermined significance), ASC-H (atypical squamous cells of undetermined significance), CIN 1–3 (cervical intraepithelial neoplasia grade 1–3).

The number of women included and excluded at each stage of the study, as well as the number of end diagnoses in cytology and histology, is presented in Fig 2. Of the 1 500 invited women, 940 (62.7%) agreed to participate in the study and received a self-sampling kit. Of these, 893 women sent a sample to the HPV laboratory. The overall participation rate was 59.5%, with a lower participation rate in the older age groups (p = 0.006). The participation rate in each age group was as follows, 62.9% (236/375) at age 60, 63.5% (238/375) at age 65, 59.5% (223/375) at age 70 and 52.3% (196/375) at age 75. Five of the women's samples contained insufficient material for the HPV assay and those women received a new self-sampling kit for resampling. All 893 samples were finally analysed.

Overall 39 women (4.4%, 95% CI 3.2–6.0) were HPV positive in the first test and 22 (2.5%, 95% CI 1.6–3.8) in the second test (Fig 2) i.e. 56.4% (22/39) were also positive in the second test collected on average 5.5 months after the first test. There was no significant difference in HPV prevalence in the different age groups (Table 1.) and the prevalence of HPV16 was 1.1% in HPV test 1. Multiple infections were found in three women in the first test (3/893, 0.3%). All HPV types tested for were found. Thirty-four women performed a second self-sample and HPV test and were included in the per-protocol approach (PP), and 19 of these women had a positive second HPV test. Of those, 17 had colposcopy and histology performed (one woman had cervix resected and one was lost to follow-up) and 76.5% (13 /17) had dysplasia (6 CIN 1 and 7 CIN 2). As the colposcopic findings were inadequate and none of the women had a fully visible transformation zone, sampling for histology by cervical abrasion and random biopsies was performed. Vaginal dysplasia was found in biopsies from iodine negative areas in the vagina. Five women did not perform a second self-sampling according to the study protocol and they were included in the ITT. Four of them had an assisted HPV test, analysed with the same PCR assay (hpVIR), at a clinic and 3 of them were HPV positive. One of them had CIN 1 and one CIN 2 in histology (Table 2). One woman also included in the ITT performed a second test by cytology and she had a CIN 3 in histology. In total, in the ITT approach, 16 women had dysplasia in histology (7 CIN 1, 8 CIN 2 and 1 CIN 3). Cytology with LBC was normal for all except one in the PP arm (ASCUS), and two in the ITT arm (ASCUS and ASC-H, respectively).

**Table 1:** HPV prevalence in the different age groups.

Age (years)	HPV pos (n)	HPV neg (n)	Total (n)	HPV pos (%)
60	10	226	236	4.2
65	12	226	238	5.0
70	10	213	223	4.5
75	7	189	196	3.6
	39	854	893	4.4

**Table 2:** Prevalence of HPV and results of cytology and histology in women HPV positive in two consecutive HPV tests.

Patient	Age	HPV test 1	HPV test 2	cytology	histology
1	60	33/52/58	33/52/58	benign	CIN 2
2	60	39	negative		
3	60	56	56	benign	CIN 2
4	60	16	16*	ASC-H	benign
5	60	16, 35	16	benign	CIN 1
6	60	18/45, 31	negative		
7	60	51	51	no sample	no sample
8	60	31, 56	31, 39, 56	ASCUS	CIN 1, VAIN 2
9	60	16	16	benign	CIN 2
10	60	39	negative		
11	65	59	negative*		
12	65	56	negative		
13	65	51	negative		
14	65	16	negative		
15	65	35	35	benign	CIN 2
16	65	33/52/58	33/52/58	benign	benign
17	65	33/52/58	33/52/58	benign	CIN 1
18	65	18/45	negative		
19	65	39	negative		
20	65	59	59	benign	CIN 1
21	65	59	negative		
22	65	16	negative		
23	70	16	negative		
24	70	18/45	18/45	benign	CIN 2
25	70	33/52/58	negative		
26	70	18/45	no sample	ASCUS	CIN 3
27	70	16, 18/45, 33/52/58	18/45, 33/52/58	benign	CIN 1
28	70	16	16, 31	benign	CIN 2
29	70	51	negative		
30	70	33/52/58	33/52/58	benign	benign
31	70	31	31	benign	benign
32	70	16	16*	benign	CIN 1
33	75	56	negative		
34	75	16	16	benign	no sample**
35	75	33/52/58	33/52/58	benign	CIN 2
36	75	31	31	benign	benign
37	75	51	negative		
38	75	31	31	benign	CIN 2
39	75	33/52/58	33/52/58, 51	benign	CIN 1

* clinical test

**cervix resected

Among the women in the PP approach, 68.4% (13/19) with a positive second HPV test had dysplasia in histology. Out of all 893 women, 16 (1.8%) had CIN, 0.8% (7/893)

CIN 1 and 1.0% (9/893) had CIN 2+ (Fig 2). In one woman with CIN 1 a vaginal lesion was found with colposcopy, and biopsy for histological evaluation showed vaginal intraepithelial dysplasia, VAIN 2. No glandular atypia was diagnosed.

The positive predictive value (PPV) for any CIN (CIN 1+) was 41.0% (16/39) after the first HPV test and 68.4% (13/19) after the second HPV test in the PP approach, and 68.2% (15/22) in the ITT approach. The PPV for CIN 2 + was 23.1% (9/39) after the first HPV test and 36.8% (7/19) after the second HPV test in the PP approach, and 36.4% % (8/22) with the ITT approach.

## Discussion

Self-sampling was well accepted among elderly women, where 59.5% of those invited participated in the study. In a recent publication based on women aged 30–49 years, where Pap-smear was compared with repeated self-sampling for HPV analysis, the participation rate was 47% in the self-sampling arm [11]. There are several studies where self-sampling has been offered to women who do not participate in the cervical cancer screening, as a strategy to increase screening coverage. As examples, there is one study from Uppsala, Sweden, with a participation rate of 39% [12] and another one from Copenhagen, Denmark, with a participation rate of 20% [22]. A higher participation rate in the current study might be explained by the fact that older women have a higher awareness of cancer risk, and are also aware that they are no longer invited to the screening programme.

An HPV prevalence of 4.4% in this age group (average age 67 years), is similar to the HPV prevalence of 4.1% found when the sample was collected from the cervix (average age 68 years) by a gynaecologist [17]. There are few studies on HPV prevalence in elderly women. In a Danish study, an HPV prevalence of 5.7% was found in women older than 65 years [23].

Although the women included in this study were from one Swedish county, we believe that data on HPV prevalence and dysplasia are generalizable to most parts of Sweden. An obvious limitation of this study is the lack of verification of possible disease among women testing HPV negative. It is, however known that the vast majority of the high-grade dysplasias are caused by oncogenic HPV and this study aimed at detecting HPV-related dysplasia. In a previous study we have data on HPV and cytology from elderly women that were HPV-negative in their second HPV test and among them the cytology was normal [17]. Loss of detection of HPV between test one and two was as high as 42.1% when the second HPV test was done on average 5.5 months after the first test. We have recently shown that the loss of detection was 37% in women aged 60–89 (average 68 years) when the time period was 3.5 months between tests one and two [17]. In a previous study on women, aged 30–65, the loss of detection was 41% with 2.7 months between tests one and two [12]. In a recent study of women aged 30–49, the loss of detection was 29% with 4.4 months between tests one and two [11]. This data indicates that the loss of detection seems to be at least as high in older women when compared with younger women. The strategy of repeating the HPV test, results in substantially fewer women requiring follow-up, and a higher specificity for the identification of CIN 2+ [12].

In line with what has been shown in other studies [16, 17], we also found a poor correlation between LBC and histology, where only 3 out of 16 with dysplasia in histology had abnormal cytology, i.e. 18.8%. This is also supported in a Danish study on women aged ≥55 years with diagnosed cervical cancer where the cervical cytology was negative in 84.6% in screening samples collected during the five years preceding cancer diagnosis [24]. This support that cytology is an inappropriate method for the detection of cervical dysplasia in this age group.

We found a CIN 2+ prevalence of 1.0% in women aged 60–75 years. This is slightly lower than that which was reported in the Swedish screening population aged 23–60 years, with a CIN 2+ prevalence of 1.3–1.4% [25]. The true prevalence of CIN 2+ is not known and it is clear that screening strategy has a great impact on the proportion of CIN identified. It was recently shown that the prevalence of CIN 2+ was 1.1% when screening was based on cytology, but 2.0% when based on HPV testing [12]. Another example is a study of women aged 56–60 where the prevalence of CIN 2+ was surprisingly low (0.2–0.3%) after screening with LBC or an HPV test with triage by LBC [26]. In a US population, the annual incidence of CIN 2/3 was 1.5 per 1 000 women screened [27].

The importance of CIN 1 in elderly women has not been studied. Several studies show that there is a high level of regression of CIN 1 in younger women but whether this is also true in the elderly is not known. According to Bekos et al, a patient's age has a considerable influence on the natural history of CIN, independent of CIN grade and HPV high-risk infection [28].

CIN 2+ is the treatment threshold for women of a fertile age, [29] but in menopausal women, this might not be optimal. The postmenopausal status, with lower oestrogen levels and immune deficiencies due to other diseases and old age, can have an impact on the risk for progression of HPV related CIN in the elderly.

Wang et al. found that Swedish women born between 1919 and 1945 who were unscreened, or screened with abnormal results in their 50s, had a relatively high risk of cervical cancer after age 60, but being screened at age 61–65 was associated with an evident risk decrease up to age 80 [30]. In women who were screened in their 50s and had only normal results, neither the cancer risk nor the risk decrease associated with screening after age 60 were sizable [30]. In our study, the oldest women were born in 1944. The change in sexual behaviour and a longer life expectancy in women born after the Second World War, are reasons to be careful not to generalise from data on women born in the first part of the twentieth century [8].

## Conclusion

Self-sampling was well accepted among elderly women, and thus constitutes a potentially appropriate strategy for cervical cancer prevention in this age group if confirmed in larger studies. We also found that among women with two HPV positive tests there was a high prevalence of CIN 2+ diagnosed by histology, which motivates screening to continue at older ages. Cytology showed extremely low sensitivity in elderly women and should therefore not be used in this age group. These data support strong preliminary evidence for a novel screening paradigm in the elderly, which must be more fully evaluated in a screening trial.
